# Time for Clarity in Exploring the Evidence and Key Concepts of Human-Centered Design in Digital Health Care: Protocol for a Scoping Review

**DOI:** 10.2196/74067

**Published:** 2025-10-20

**Authors:** Luisa Mejia, Björn Bergh, Björn Schreiweis

**Affiliations:** 1 Institute for Medical Informatics and Statistics, Kiel University and University Hospital Schleswig-Holstein Kiel Germany

**Keywords:** patient-centric health care, user experience, user interface, usability, mobile health, mHealth human-centered design, design thinking, scoping review, participatory design, generative artificial intelligence, GenAI

## Abstract

**Background:**

Human-centered design (HCD) methodologies such as design thinking (DT), user-centered design, cocreation, and participatory design (PD) have been adopted to facilitate user and stakeholder involvement in the development of eHealth applications. However, there is frequent confusion around these methodologies, leading to the fragmentation of the discourse and limited integration opportunities. The absence of an empirically grounded framework for HCD limits research and theoretical consensus, particularly in the highly regulated context of eHealth solution development. For this scoping review, the term HCD will be used as an umbrella term, under which the terms user-centered design, patient-centered design, cocreation, co-design, PD, and DT will be used in this protocol.

**Objective:**

In this paper, we describe a protocol for a scoping review that aims to explore and analyze the scope, definitions, key concepts, and motivations reported in peer-reviewed studies that have applied stakeholder engagement methods such as HCD, PD, or DT in developing eHealth applications.

**Methods:**

A team of 3 reviewers will conduct this scoping review to identify and synthesize key concepts at the intersection of HCD methodologies and their application to the development of eHealth applications. We will follow the Joanna Briggs Institute methodology for scoping reviews and the guidelines for conducting systematic mapping studies in software engineering. The reporting of the results will be guided by the PRISMA-ScR (Preferred Reporting Items for Systematic Reviews and Meta-Analyses Extension for Scoping Reviews) extension. This review will include only primary studies reporting on the experience, challenges, and applicability of HCD for the design and development of eHealth applications, identified through the PubMed, IEEE Xplore, Web of Science, Scopus, PsycINFO, CINAHL, and ACM Digital Library databases, and limited to articles from the past 10 years.

**Results:**

A preliminary search applying the search strategy resulted in 826 records. The search was initiated in March 2025. Title and abstract screening will conclude by mid-2025, followed by full-text screening, data extraction, and analysis in the second half of 2025. Results are expected to be submitted for publication in the first half of 2026.

**Conclusions:**

This protocol describes a systematic approach for conducting a scoping literature review, aimed at synthesizing definitions, concepts, and methodologies related to HCD in eHealth application development. This review aims to identify research gaps and trends to guide the future of mobile health innovation, with a focus on improving adoption and long-term sustainability, particularly from the perspective of technologies for vulnerable populations.

**International Registered Report Identifier (IRRID):**

DERR1-10.2196/74067

## Introduction

### Background

According to a report by the Institute of Medicine, today’s health providers have access to more research findings and more technology than ever before. However, there are serious concerns about the inadequate quality of health care, driven by four key factors: (1) the growing complexity of science and technology, (2) the increase in prevalence of chronic conditions as people live longer, (3) a decentralized, inefficient health care delivery system, and (4) challenges in leveraging information technology, as patients increasingly seek health-related information and advice online [1]. Human-centricity, which emphasizes health care solutions that are humane and respectful of the needs and preferences of individuals, is 1 of 6 key factors needed to close this quality gap in the new era of health care [1].

### Adoption of eHealth Solutions

When developed correctly, eHealth applications can facilitate self-care promotion, enable informed decision-making, foster patient engagement and empowerment, and ultimately improve patients’ satisfaction and health outcomes [2,3]. eHealth solutions entail various digital technologies for health management and care and may include telehealth applications, such as videoconferencing for medical consultations; electronic health records for storing, managing, and accessing patient health information; mobile health (mHealth) apps, such as medication reminders, diabetes self-management, mental health apps, and artificial intelligence (AI) algorithms [4,5].

Despite their potential, the rapid and costly development of digital health technologies frequently leaves no space for user involvement in the design process, leading to solutions that fail to address real-world needs [6,7]. Consequently, low adoption rates result in significant technological waste [8-12].

Moreover, patient expectations for eHealth solutions are very high, as users expect these applications to provide a similar user experience to their shopping, messaging, or banking solutions. However, the low adoption rates demonstrate that eHealth applications still have a steep path ahead [11]. Similarly, health care professionals expect eHealth applications to integrate into their workflow easily, allowing more time for patient care [12]. In reality, many health care professionals spend large amounts of their time working with IT systems that do not add value to the quality of care [13,14].

### Methods for Stakeholder Engagement: Design Thinking, Human-Centered Design, and Participatory Design

The importance of human-centricity—placing patients, caregivers, and health care professionals (ie, physicians, nurses, and other medical staff) at the heart of the development process [15-17] to truly meet their needs and expectations—is acknowledged but rarely applied to eHealth applications [18]. Methods such as human-centered design (HCD), design thinking (DT), cocreation, and participatory design (PD) have been adopted in other industries to address this issue by helping software developers incorporate the needs, experiences, and feedback of end users and stakeholders in the development process of digital applications in general. A recent systematic literature review by An et al [19] reported studies of eHealth applications comparing traditional interventions to HCD interventions, where the latter showed greater satisfaction, usability, and effectiveness.

### HCD Approach

The term HCD has been defined in the International Organization for Standardization (ISO) standard 9241–220 as an approach to systems design and development that aims to make interactive systems more usable by focusing on the understanding of interactions among humans and other elements of a system to optimize human well-being and overall system performance [20]. The term *HCD* goes beyond *user-centered design (UCD)* to foster the involvement of other stakeholders, not only those typically considered users, throughout design and development activities [6,21].

### DT Methodology

The DT methodology is an iterative, nonlinear, and human-centered method that helps incorporate users’ needs and feedback throughout the development process. DT has been recognized as a problem-solving methodology and a driver of innovation and change [22-25]. According to Stanford school, the 5 phases of the process are empathy, define, ideate, prototype, and test [26].

On a practical level, DT has been interpreted as a trilogy of interlinked modes: (1) a process with a sequence of steps, (2) a toolbox with a collection of methods, and (3) a mindset with a set of human-centered principles [27,28].

Over the last 40 years, researchers at Stanford University have studied this methodology, which has spread to other universities, such as Aalto (Finland), Potsdam (Germany), and St Gallen (Switzerland). It has been adopted in the private sector across many industries such as business, fintech, hospitality, education, and aviation, to solve complex problems [22,29,30]. Examples of adopters are major companies such as Deutsche Bank, Procter & Gamble, Google, Apple, and SAP [18]. More recently, DT has emerged as a critical tool to drive innovation in health care [5,6,19,22,31-35]; however, when considering the scope of all ongoing research, development, and innovation in health care, DT has been applied sparsely [12,20].

### Cocreation and Co-Design

A core principle of DT is radical collaboration, also known as cocreation, which emphasizes trust-building within design teams and among stakeholders, ensuring that all participants actively contribute and learn from one another throughout the design process [36,37]. This principle relies on flexibility in thinking, creativity, and openness to new ideas and perspectives [38].

### PD Approach

PD has been defined as a human-centric approach to technology development guided by the enhancement of workplace democracy, mutual learning, and empowerment [12], and as a toolbox for engaging users to deliver better products [24]. The principles of stakeholder participation are aligned with participatory ergonomics and other PD methods, such as experience-based design [39].

### A Wicked Problem

The DT method has been criticized for lacking theoretical and methodological rigor [24,40,41]. The literature often presents DT as vague and sometimes ambiguous [42] or as “a practice rather than a precise science” [43]. Other descriptions and theoretical foundations provide a more specific definition of DT as a methodology suited for use in broad and multidisciplinary settings [23,25,44-52]. Furthermore, there is often confusion between DT, HCD, UCD, PD, patient-centered design, gamification, and agile methods [30], which leads to the fragmentation of the discourse and missing opportunities for integration. The absence of an empirically grounded framework for HCD limits research and theoretical consensus, particularly in the highly regulated context of the development of eHealth applications [23,53-55].

Several studies report (1) the lack of coherent evidence- and theory-driven methodologies that promote user and stakeholder involvement in the development process of eHealth applications [5,21-24] and (2) the need for more cocreation between vendors and stakeholders in the development process of eHealth applications [56,57]. These 2 conditions are paramount factors for adoption. They highlight the need to clarify the key concepts and key characteristics related to the methods promoting human-centricity and stakeholder engagement.

HCD methodologies can be particularly valuable in the context of eHealth applications, and their use is expected to increase, as they ensure that the software solutions are not only technically robust but also focused on user needs and real-world contexts, leading to more effective and widely adopted developments [19,22,34]. Conversely, the overall understanding and specific steps on how the HCD processes are applied in complex and highly regulated environments, such as digital health care, remain underexplored. Furthermore, the empirical evidence on the application of the methods within the eHealth sector is surprisingly limited in comparison to other industries [5,58,59], especially concerning solutions for vulnerable patient groups [60].

### Goal of This Review

This scoping review will act as a stepping stone in developing a methodological framework by (1) characterizing the extent to which primary studies reported in peer-reviewed articles that are publicly available in the literature have adopted HCD for the development of eHealth applications and (2) mapping the quantitative and qualitative evidence to shed light on the methods, tools, and principles that have been used in the context of HCD approaches to inform the development of eHealth applications.

Our goal with this review is to reduce ambiguity around the concepts of HCD in the context of the software development of digital health applications and to empower researchers to develop solutions that place the needs of users and stakeholders at the forefront instead of being ”lost in translation” amid the confusion of the terms, while maintaining compliance.

### Review Questions

Guided by the ultimate goal of creating a methodological framework, this review explores *what key concepts and instruments of HCD are available and how they are being applied in the context of the software development of eHealth applications*. This high-level question is broken down into 4 specific review questions (Table 1).

**Table 1:** Review questions (RQs).

RQs	Subquestions
RQ1. Concept: what is HCD^a^?	Which theoretical frameworks, key concepts, and terms of HCD have been used in the reported studies?
RQ2. Process: in what ways is HCD being used?	Which HCD tools, processes, and mindsets have been applied in the studies, and what approaches have been used to evaluate the application of HCD methodologies?
RQ3. Motivation: why is HCD applied?	Why do teams choose HCD methodologies over other methodologies?
RQ4. General: where, when, and who is applying the method?	In which geographical locations (based on author affiliation and domains of health care) have the studies applying the HCD methodologies been published?

^a^HCD: human-centered design.

## Methods

### Overview

We have chosen to conduct a scoping review of the literature, which is a systematic and effective method for identifying and synthesizing key concepts and methods in the literature on a given topic, with the purpose of summarizing and mapping the available evidence. Unlike traditional systematic reviews, scoping reviews do not aim to produce a critical appraisal of the literature but rather to provide an overview or map of the available evidence [61].

This scoping review will follow the Joanna Briggs Institute methodology for scoping reviews [62] and the guidelines for conducting systematic mapping studies in software engineering [63]. This protocol consists of 5 stages: (1) defining the review question; (2) identifying relevant studies or developing a search strategy; (3) study screening and selection; (4) extracting and charting the data from the studies included; and (5) collating, summarizing, and reporting the data.

The reporting of the results will be guided by the PRISMA-ScR (Preferred Reporting Items for Systematic Reviews and Meta-Analyses Extension for Scoping Reviews).

### Search Strategy

The studies will be identified initially through searches of the following 7 online databases: PubMed, IEEE Xplore, the Web of Science Core Collection, Scopus, APA PsycINFO, CINAHL, and the ACM Digital Library. These databases were considered because they cover the technical and clinical literature, as well as aspects such as psychology, design, human-computer interaction (HCI), and implementation science, reflecting the multidisciplinary nature of the literature.

Key concepts and domains were identified based on a preliminary literature review of key articles known to the authors. The keywords contained in the abstracts and titles led to several search terms that were clustered into three main themes, following the concept and context outlined in the inclusion criteria: (1) HCD, (2) eHealth, and (3) technology and development. The related key concepts for the 3 themes are presented in Table 2. These key terms will serve as the basis for the search in each of the databases.

**Table 2:** Search strategy and key terms.

Domain and context	Search strings^a^	Controlled terms
Human-centered design	(“Design Thinking” OR “Human Centered Design” OR “User Centered Design”[MeSH^b^] OR “Co-design” OR “Participatory Design” OR “Service Design”)	“User-Centered Design”[MeSH]
eHealth	(“eHealth” OR “telemedicine” [MeSH] OR “mHealth” OR “Health Informatics” [MeSH] OR “digital health” OR “remote monitoring”)	“Telemedicine” [MeSH]; “Health Informatics” [MeSH]; “Digital Health” [MeSH]
Technology and development	(“Software Design” [MeSH] OR “User Experience” OR “technology development” OR “user requirements” OR “Digital Technology” [MeSH]	“Digital Technology” [MeSH]; “Software Design” [MeSH]

^a^All domain search strings were combined using the Boolean operator AND.

^b^MeSH: Medical Subject Headings.

The search terms have been tested term by term for relevance and performance in PubMed. We tested the search results by adding and removing search terms to identify the most relevant ones, observing the number of results added by a specific term to the results, and determining the versions of the search that should be moved forward for relative recall testing. Terms that did not add any unique results were removed to streamline the search terms.

For additional validation, different versions of the search strategy were created to test the performance of a broad versus narrow interpretation of the terminology and the best-suited PubMed field tags (eg, [ti], [tiab], [tw]). Ultimately, each search term was tested multiple times to determine the types of studies returned before being included in the final search terms. The final search string has been peer reviewed by a librarian.

Due to time and resource limitations, articles will not be screened for additional studies (snowballing). The search will be limited to the last 10 years of publication.

### Inclusion Criteria

#### Population

No restrictions are applied to participants in this scoping review.

#### Concept

The object of this scoping review will be the exploration of HCD methodologies, with a focus on DT as a methodology to design, develop, describe, report, or document experiences during the software development of eHealth applications. Articles will be included if two premises are fulfilled: (1) the research article has the main focus on conceptualizing HCD methodologies, either through definition, descriptive methods, attributes, or relationships, and (2) the concepts are used in relation to the development of software applications in the area of eHealth, medical informatics, and digital health care.

#### Context

The studies considered for this review will include studies in all health care settings and areas of medicine in the private or public sector, as long as the eHealth applications are intended for medical purposes, such as diagnosis, prevention, monitoring, therapy, or rehabilitation. However, studies reporting on the development of programs or education in the broader sense of health care will not be included in the review.

#### Types of Evidence Sources

This review will include all peer-reviewed primary research articles that apply and report on the application of HCD and methods that have used the HCD methodology for eHealth applications. The review will not include any peer-reviewed methodology articles, literature reviews, meta-analyses, guidelines, or opinion articles reporting on HCD.

Furthermore, articles published between 2015 and 2025 in English, Spanish, and German will be included, as LM is fluent in these languages. An overview of the inclusion and exclusion criteria is provided in Textbox 1.

**Inclusion and exclusion criteria.**

**Inclusion criteria**

- Peer-reviewed articles, including conference proceedings, published in open-access journals or journals accessible to the author
- Primary studies or empirical studies reporting on experiences, challenges, applicability of human-centered design (HCD) for the design and development of eHealth applications
- Written in English, German, or Spanish
- Published between 2015 and 2025

**Exclusion criteria**

- No abstract available
- No full text available
- Published in a language other than German, English, or Spanish
- Literature reviews, methodological articles, opinion articles, theoretical articles, or meta-analyses
- Studies already evaluated in one of the reviewed articles (duplicates)
- Studies in which HCD is not mentioned in the title or abstract, not conceptualized, not clearly operationalized, or lacking a theoretical underpinning
- Not relevant to medical informatics or digital health

### Study Screening and Selection

On the basis of the inclusion criteria in Textbox 1, all retrieved articles will be organized using the bibliographic reference management software Zotero, and any duplicates will be removed. Zotero will be used for the initial screening of titles and abstracts. Selected articles will be imported into the software for qualitative and mixed methods analysis, and MaxQDA [63] will be used for full-text analysis.

To limit the risk of selection bias and interpretation bias, we will use a triangulation approach in which 3 reviewers will be involved in the screening and adjudication process. Two reviewers, 1 human and 1 AI robot screener (Nested Knowledge [64]), will independently screen the data. The AI robot screener (second reviewer) will be trained with a set of 50% of the records to ensure accuracy. A third reviewer (a human expert in medical informatics) will compare the robot screening results to the screening decisions of the first reviewer (human) and will adjudicate any conflicts.

As an additional layer to limit interpretative bias, the third reviewer will randomly select 30% of the included studies for an additional independent review of the screening process, ensuring consistency and reliability in data extraction. In case of disagreement, the corresponding article will be reviewed by a fourth reviewer (an expert in medical informatics), who will act as a mediator.

Careful record keeping will be maintained through a standardized screening table documenting reasons for rejection, which will be used by all authors to minimize potential bias and allow for transparency. This table will be made publicly available as part of the results publication. Reasons for exclusion of the full-text articles will be documented and reported in the scoping review in a PRISMA (Preferred Reporting Items for Systematic Reviews and Meta-Analyses) flow diagram. To ensure consistent and accurate assessments and the performance of the AI model, statistical parameters such as interrater reliability, recall, and accuracy will be evaluated and reported.

### Data Extraction and Charting

A data charting table, including general study characteristics and variables related to the concept, context, review questions, and key concepts, will be included in the review.

To validate the completeness and accuracy of the data charting table, a pilot extraction process will be conducted for 10 of the selected articles. The data will be extracted in parallel by the 2 human reviewers, and any uncertainties that arise will be discussed. If necessary, the data charting table will be adjusted accordingly.

After validating the data charting table, the full charting of the data will be done by 1 reviewer with random quality checks by the second reviewer, who will review 30% of randomly selected articles and compare them to the results obtained by the first reviewer to ensure consistency. Any uncertainties or disagreements will be discussed between the 2 reviewers until consensus is reached. If a decision cannot be made, a third reviewer will act as a mediator. The data charting table will be made publicly available as part of the results publication.

On the basis of the prereviewed literature and acknowledging the ambiguity surrounding HCD terminology, the authors will adopt a deductive approach, deriving subcodes from the selected studies and structuring them within a framework. The main codes will be generated inductively, aligned with the predefined review questions (Table 1). Careful record keeping will be supported through the standardized charting table, which will be used by all authors to minimize potential bias.

Examples of items to be included in the data charting table are: (1) general characteristics of the study (eg title, year, and country), (2) concept (terms and theories used in connection with HCD in the reported studies), (3) process (tools, mindset and life cycle of HCD applied in the reported studies), (4) motivation (why teams choose HCD methodologies over other methodologies); and (5) evaluation (which practices have been used to evaluate the application of HCD methods in the reported studies)

### Data Collating, Summarizing, and Reporting

This review will follow a mixed methods approach. The data will be analyzed using descriptive statistics for all quantitative data items, such as sample size, sample demographics, and the methodological approach. The data items of a qualitative nature will be analyzed using the methodology developed by Mayring for qualitative content analysis, which aims to systematically code and organize the data based on a structured coding framework consisting of categories that can be generated deductively and inductively [64].

The information for each extracted field will be tabulated, grouped by themes and subthemes, and illustrated visually. To create a compelling presentation of the findings, the data will be available in tabular form, accompanied by graphic visualizations in the form of evidence maps where possible, and a narrative summary.

## Results

A preliminary search applying the key terms (Table 2) in all 7 databases resulted in 826 records. The search was initiated in March 2025. Title and abstract screening will conclude by mid-2025, followed by full-text screening, data extraction, and analysis in the second half of 2025. The results are expected to be submitted for publication in the first half of 2026.

## Discussion

### Anticipated Findings

This protocol outlines a systematic approach for conducting a rigorous scoping literature review, aimed at summarizing the current evidence in the form of evidence maps, helping readers identify where evidence exists and where gaps remain, and understanding the terms, definitions, and methodologies of HCD and related participatory approaches in the development of eHealth technologies. This knowledge may help reduce confusion, facilitate interdisciplinary collaboration, and support the implementation of HCD methodologies.

We anticipate identifying patterns in how these methodologies are applied across different phases of the development process, target populations, and health domains. A particular focus will be placed on understanding whether and how these methods are used in the context of vulnerable or underserved populations, such as children, older adults, or individuals with cognitive or mental health conditions.

We expect to identify research gaps and trends that will inform the current and future development of mHealth technologies to enhance their adoption and sustainability.

### Comparison to Prior Work

Several studies have examined the role of HCD and other stakeholder engagement methodologies in health care. Kauppinen et al [5] investigated the potential of HCD to enhance the benefits of health care software projects; the results of this review are pending. Jacob et al [65] synthesized the literature on patient adoption of health applications, emphasizing the importance of sociotechnical factors, inclusive design, integration into the patient journey, and comprehensive education and support. Leary et al [38] reported that clinical providers should be proficient in DT to create effective health interventions, noting inconsistencies in its application. Nimmanterdwong et al [66] demonstrated that HCD can yield positive outcomes for older adults while highlighting the need for more rigorous, goal-oriented studies in mHealth for this population. Altman et al [22] found HCD-based interventions to be more usable and effective than conventional approaches, showing that methodological and quality limitations are barriers to their implementation. An et al [19] found that DT can produce usable, acceptable, and effective mHealth applications and recommended standardizing HCD practices to improve sustainability and integration into development processes.

In summary, this scoping literature review builds on previous research by exploring how HCD methodologies have been implemented to develop eHealth applications. While earlier work in the field often focused on a single methodology or specific health condition or technology, few studies have offered a comprehensive overview of the diverse HCD-related approaches used in eHealth. Our review adopts a broader methodological spectrum, encompassing UCD, patient-centered design, cocreation, co-design, PD, and DT. Furthermore, by including work from multiple disciplines, such as health informatics, HCI, and implementation science, we aim to complement the existing literature by identifying both common practices and underexplored areas in the design of human-centered digital health solutions.

### Strengths and Limitations

A key strength of this protocol is the use of a comprehensive and interdisciplinary search strategy, incorporating both controlled vocabulary (eg, Medical Subject Headings [MeSH] terms) and free-text keywords across multiple databases spanning health, psychology, and computer science. Despite efforts to balance sensitivity and precision, the broad scope of terms such as “technology,” “DT” [50,52] or “user experience” may result in the inclusion of studies only marginally relevant to HCD in eHealth.

One limitation of this study is that, as with all scoping reviews, the lack of methodological quality appraisal may limit the ability to make definitive claims about the effectiveness of specific approaches. In addition, due to time constraints, backward and forward citation tracking will not be performed.

LM will lead the data collection and analysis processes, is trilingual in Spanish, English, and German, and self-identified at the time of drafting this manuscript as a woman and an ethnic minority scholar of Latin American origin living in Germany. BS self-identified as a Caucasian male born and raised in Germany; he speaks German, English, Swedish, and French; and contributed to refining the theoretical framework. LM and BS contributed to conceptualizing the study. However, it is likely that our backgrounds may influence the interpretations of the data. To minimize this bias, LM and BS will make efforts to take notes on all preconceptions that arise during study inclusion in order to limit these existing assumptions during the data collection and analysis processes.

### Future Directions

The findings of this scoping review may inform the development of best practice guidelines for researchers and practitioners seeking to apply HCD methods in digital health. They may also point to the need for greater methodological transparency and interdisciplinary collaboration in this domain. We expect that this review will highlight important gaps in current research, such as the limited inclusion of vulnerable populations in co-design processes or the inconsistent reporting of HCD methods. This paper is part of a larger research project, and more specific research activities will be described in separate papers.

### Dissemination Plans

We plan to disseminate the final results through multiple channels. In addition to submitting the findings for open-access publication in a peer-reviewed journal, we will present the results at relevant conferences in digital health, HCI, and implementation research. The findings will also be shared with design and clinical stakeholders through webinars, workshops, and summaries tailored to practice-oriented audiences.

## Data Availability

Data sharing is not applicable to this study as no datasets were generated or analyzed during the preparation of this manuscript.

## Abbreviations

AI: artificial intelligence
DT: design thinking
HCD: human-centered design
HCI: human-computer interaction
ISO: International Organization for Standardization
MeSH: Medical Subject Headings
mHealth: mobile health
PD: participatory design
PRISMA: Preferred Reporting Items for Systematic Reviews and Meta-Analyses
PRISMA-ScR: Preferred Reporting Items for Systematic Reviews and Meta-Analyses Extension for Scoping Reviews
UCD: user-centered design
